# Chemoenzymatic conversion of glycerol to lactic acid and glycolic acid

**DOI:** 10.1186/s40643-022-00561-z

**Published:** 2022-07-18

**Authors:** Yue Ma, Tianzhen Li, Zijian Tan, Long Ma, Haifeng Liu, Leilei Zhu

**Affiliations:** 1 State Key Laboratory of Food Nutrition and Safety, College of Biotechnology, Tianjin University of Science and Technology, No 9, 13th, Avenue, Tianjin Economic and Technological Development Area (TEDA), Tianjin, 300457 China; 2 Tianjin Institute of Industrial Biotechnology, Chinese Academy of Sciences, National Technology Innovation Center of Synthetic Biology, 32 West 7th Avenue, Tianjin Airport Economic Area, Tianjin, 300308 China; 3 Jiangsu Collaborative Innovation Centre of Chinese Medicinal Resources, Industrialization, School of Pharmacy, Nanjing University of Chinese Medicine, 138 Xianlin Rd, Nanjing, 210023 Jiangsu China

**Keywords:** Chemoenzymatic catalysis, Glycerol, Dihydroxyacetone, Lactic acid, Glycolic acid

## Abstract

Catalytic valorization of raw glycerol derived from biodiesel into high-value chemicals has attracted great attention. Here, we report chemoenzymatic cascade reactions that convert glycerol to lactic acid and glycolic acid. In the enzymatic step, a coenzyme recycling system was developed to convert glycerol into 1,3-dihydroxyacetone (DHA) with a yield of 92.3% in potassium phosphate buffer (300 mM, pH 7.1) containing 100 mM glycerol, 2 mM NAD^+^, 242 U/mL glycerol dehydrogenase-GldA and NADH oxidase-SpNox^K184R^ at 30 °C. Subsequently, NaOH or NaClO_2_ catalyzes the formation of lactic acid and glycolic acid from DHA. The high yield of lactic acid (72.3%) and glycolic acid (78.2%) verify the benefit of the chemoenzymatic approaches.

**Graphical Abstract:**

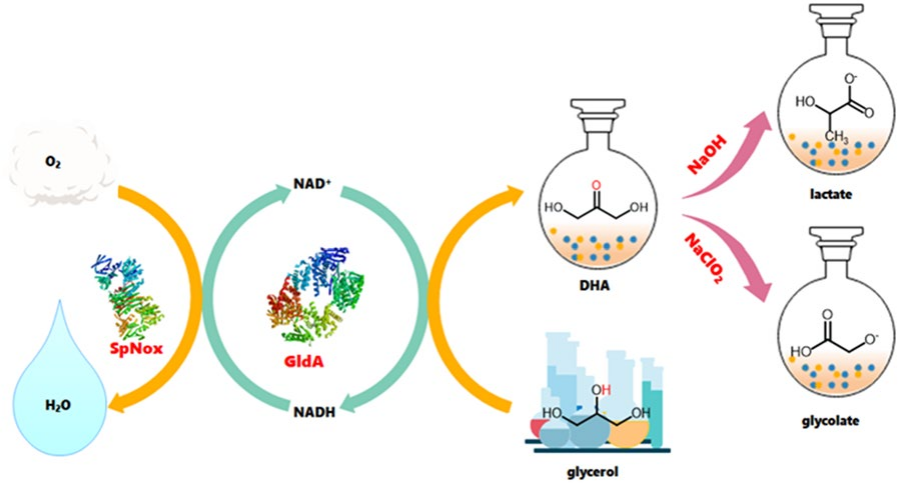

**Supplementary Information:**

The online version contains supplementary material available at 10.1186/s40643-022-00561-z.

## Introduction

With the urgency of reducing the dependence on fossil fuels, (Kosamia et al. 2020) the fuel energy and chemical commodities is increasingly preferred towards the use of clean liquid biofuels, ethanol and biodiesel. However, large amount of crude glycerol was produced as a by-product accounting for 10% of the produced biodiesel (Veluturla et al. 2018), with an estimated amount of 4 × 10^6^ tons by 2024 (Singh and Ahuja 2018). Moreover, since glycerol is a by-product with low economic value and cannot be safely disposed in the environment, the generated excess glycerol may become an environmental threat. Therefore, it is highly profitable to transform low-value but versatile glycerol to high value-added chemicals (Walgode et al. 2020). Glycerol is an important C3 platform compounds (Liu et al. 2019) that can be upgraded into various chemical building blocks through chemical oxidation, reduction (Xu and Ni 2015), dehydration, deoxydehydration reaction or a combination of the aforementioned methods, e.g., to allyl alcohol (Tshibalonza and Monbaliu 2017), acrylic acid (Sun et al. 2017), divinyl glycol (Sotto et al. 2016), DHA (Lari et al. 2015), etc.

The conversion of glycerol to DHA was performed by glycerol dehydrogenase (EC 1.1.1.6, EC 1.1.1.72, EC 1.1.99.22). Glycerol dehydrogenase-GldA (from *Escherichia coli*, EC 1.1.1.6) is an enzyme present in bacterial cytoplasm, converts glycerol to 1,3-dihydroxyacetone accompanied by NAD^+^ to NADH. It should be noted that it catalyzes the reaction of glycerol for a reversible reaction, glycerol oxidation in alkaline conditions and DHA reduction in acidic conditions. Therefore, it is necessary to pay attention to the change of reaction pH when using it for experiments. For the cofactor regeneration, often NADH oxidase which catalyzes the oxidation of NADH by reducing O_2_ to H_2_O through four-electron reduction was used.

Lactic acid and glycolic acid are important building blocks of biodegradable polymer, poly-lactic acid (PLA), poly-glycolic acid (PGA) and poly lactic-co-glycolic acid (PLGA). Lactic acid (López-Gómez et al. 2019) is a three carbon alpha hydroxycarboxylic acid widely used in food, medicine, cosmetics, detergents and dairy industries (Marianou et al. 2019) as well as the synthesis of polylactic acid (Huang et al. 2021), lactate ester (Hasegawa et al. 2008), pyruvate (Liu et al. 2010), acrylic acid (Wang et al. 2008), 1,2-propanediol (Oude Elferink et al. 2001), 2,3-pentanedione (Niu et al. 2019), etc. The production of lactic acid by microbial fermentation is the main production method (Wang et al. 2020), because of the wide source of raw materials, low production cost, high optical purity and high safety. Currently, more than 90% of the global lactic acid production is made by the microbial fermentation (Zhang et al. 2019). Arabinose, pentose, sucrose and lignocellulosic hydrolysates have been used as substrates for microbial fermentation of lactic acid. Glycolic acid is a two-carbon α-hydroxy acid, which is widely used in pharmaceutical, textile and food. It is often used in emulsion polymers, solvents and additives in inks and paints to improve flow performance and gloss (Lasprilla et al. 2012). Furthermore, glycolic acid can copolymerize with lactic acid to form poly PLGA, which is an important biodegradable material with unique properties such as non-toxic, biocompatible and degradable in vivo (Shi et al. 2006). PLGA is widely used in the fields of medical polymers such as suture and tonic materials, surgical sutures, drug sustained-release carriers, tissue engineering, etc. (Huang et al. 2020). Glycolic acid is mainly produced by chemical processes, e.g., carbonylation of formaldehyde with synthesis gas (Shi et al. 2020b) or treatment of carbon monoxide with water (Shi et al. 2020a), electrolytic reduction of oxalic acid and hydrolysis of glycolonitrile (Zhu et al. 2020). Microbial fermentation with engineered metabolic pathways have been developed for the production of glycolic acid. Using the lack of a functional carbon-concentrating mechanism (CCM), glycolic acid was accumulated through an inefficient and incomplete photorespiratory pathway (Yun Eun et al. 2021). Using glucose as substrate, glycolysis pathway was integrated with glycolic acid pathway, and finally the yield of glycolic acid with the potency of 41 g/L/h was achieved (Zhu et al. 2021). The titer of glycolic acid in 5-L bioreactor reached 40.9 g/L after multiple rounds of strain engineering using biosensor-based high-throughput method (Xu et al. 2021). Chemical method is commonly used in industrial production of glycolic acid (Shi et al. 2019). Chemoenzymatic cascade reactions are synergistic tandem reactions by combining a variety of chemical catalysts with biocatalysts in vitro, which can effectively reduce by-products and wastes and make the products easier to be purified (Luo et al. 2021).

Inspired by a previous report on lactic acid generation through chemical catalysis (Li et al. 2020), such as KOH, Ba(OH)_2_, Ca(OH)_2_ (Lux and Siebenhofer 2013), DHA was firstly dehydrated to pyruvic aldehyde and then isomerized to lactic acid via the intramolecular Cannizzaro reaction under alkaline conditions (Evans and Cornthwaite 2002). And the C–C bond of DHA was selectively cleaved and then oxidized to glycolic acid and formate in the presence of H_2_O_2_ and NH_4_OH (Pullanikat et al. 2010). We decided to use NaOH as catalysts for the conversion of DHA into lactic acid, and NaClO_2_ as oxidant for the oxidation of DHA to glycolic acid.

In this study, two-step chemoenzymatic cascade reactions were developed to convert glycerol to lactic acid and glycolic acid, respectively. Reaction conditions were optimized to achieve desirable yield. This is a novel pathway for converting glycerol to lactic acid and glycolic acid.

## Results and discussion

### Design of the cascade reactions

**Scheme 1:**
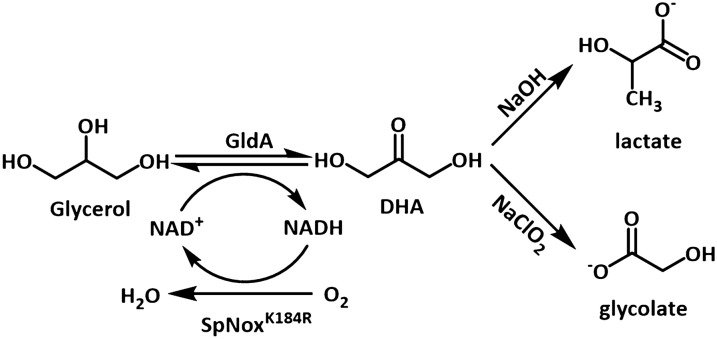
A diagram of the cascade reaction in this experiment

As shown in Scheme 1, the cascade reaction starts with the glycerol dehydrogenase (GldA) catalyzed oxidation of glycerol into DHA, which is subsequently transformed to lactic acid and glycolic acid in different chemical reaction systems. For the generation of lactic acid, NaOH was used to promote the rearrangement of DHA into lactic acid where DHA was firstly dehydrated to pyruvic aldehyde and then isomerized to lactate via the intramolecular Cannizzaro reaction under alkaline conditions. For the generation of glycolic acid, DHA was oxidized to glycolate acid by NaClO_2_.

### Enzymatic conversion of glycerol to DHA

For the initial step of this cascade, in vitro biotransformation of glycerol to DHA was achieved with glycerol dehydrogenase-GldA (Gene ID: CP034658.1) (from *Escherichia coli*) (Piattoni et al. 2013) using NAD^+^ as a cofactor and the NADH oxidase-SpNox^K184R^ (from *Streptococcus pyogenes*) (Oscar; and Jan-E 2015) for NAD^+^ regeneration, which requires only O_2_ as the co-substrate. The GldA gene was cloned into T7 promoter-based plasmid pET21b to construct pET21b-GldA. The SpNox gene with an amino acid substitution K184R was synthesized and cloned into the pRSFDuet-1. pET21b-GldA and pRSFDuet-SpNox^K184R^ were expressed in *E. coli*/BL21-Gold (DE3), respectively. After the strain was cultured and expressed, the protein was collected from the broken strain and then for the following purification.

**Fig. 1:**
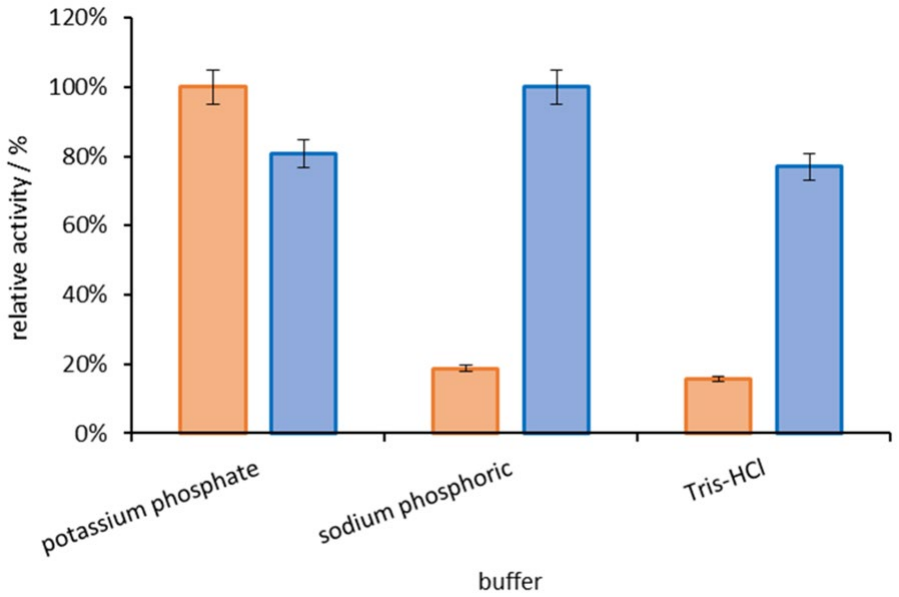
The activity of GldA (orange bar) and SpNox^K184R^ (blue bar) with different kinds of buffer

Firstly, three kinds of buffer solution (potassium phosphate buffer (100 mM K_2_HPO_4_ and KH_2_PO_4_, pH7.1), sodium phosphoric buffer (100 mM NaH_2_PO_4_ and Na_2_HPO_4_, pH7.1), Tris–HCl buffer (100 mM Tris–HCl, pH7.1)) were investigated for the oxidation of glycerol catalyzed by dehydrogenase-GldA and NADH oxidase-SpNox^K184R^ (Xu et al. 2017). Enzyme activity of GldA and SpNox^K184R^ was determined by the slop of decrease and increase of NADH observed at 340 nm, 30 °C. As shown in Fig. 1, glycerol dehydrogenase-GldA exhibited acceptable activity in potassium phosphate buffer, but nearly inactive in Tris–HCl buffer. Meanwhile, SpNox^K184R^ showed acceptable activity in Tris–HCl buffer and potassium phosphate buffer. Considering the compatibility of these two enzymes in the same reaction system, potassium phosphate buffer was selected for the GldA-SpNox^K184R^ coupled enzyme system for further investigation.

**Fig. 2:**
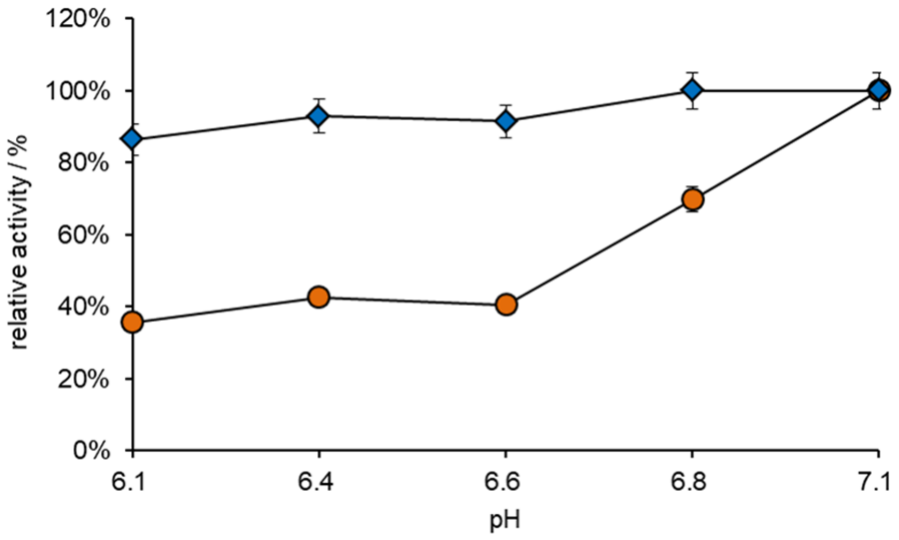
The activity of GldA (orange circle) and SpNox^K184R^ (blue square) at different pH, ranging from 6.1 to 7.1 (100 mM K_2_HPO_4_ and KH_2_PO_4_)

After the selection of the buffer solution, the optimum pH value of GldA and SpNox^K184R^ were investigated with the potassium phosphate buffer, ranging from pH 6.1–7.1 (Fig. 2). The activity of GldA and SpNox^K184R^ was gradually increased with the elevated pH value. Since GldA and SpNox^K184R^ can reach highest activity at pH 7.1, the following experiments were carried out at pH7.1.

**Fig. 3:**
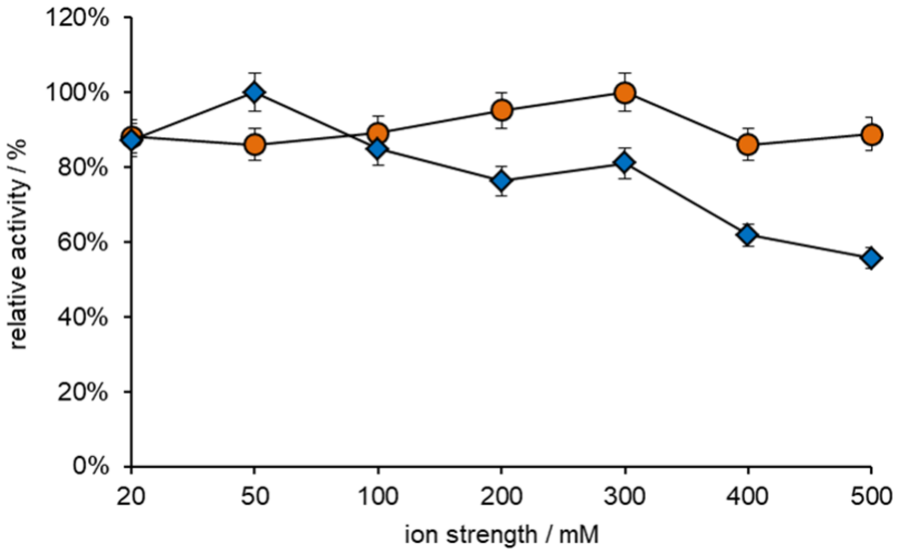
The activity of GldA (orange circle) and SpNox^K184R^ (blue square) in different concentrations of pH 7.1 potassium phosphate buffer

The concentration of the potassium phosphate buffer was optimized for the optimum activity of GldA and SpNox^K184R^, ranging from 20 to 500 mM. Figure 3 shows that 300 mM is the optimal concentration of potassium phosphate buffer for GldA and 50 mM was the optimal concentration for SpNox^K184R^. On overall consideration, as a key enzyme, GldA's activity affects the catalytic efficiency of the whole system, so 300 mM potassium phosphate buffer is selected for subsequent experiments.

The enzymatic activity of GldA and SpNox^K184R^ were determined to be 24.2 U mg^−1^ and 34.62 U mg^−1^, respectively. One unit of the enzyme activity was defined as catalyzing the conversion of 1 μmol NADH to NAD^+^ per minute under standard conditions for GldA or vice versa for SpNox^K184R^.

**Fig. 4:**
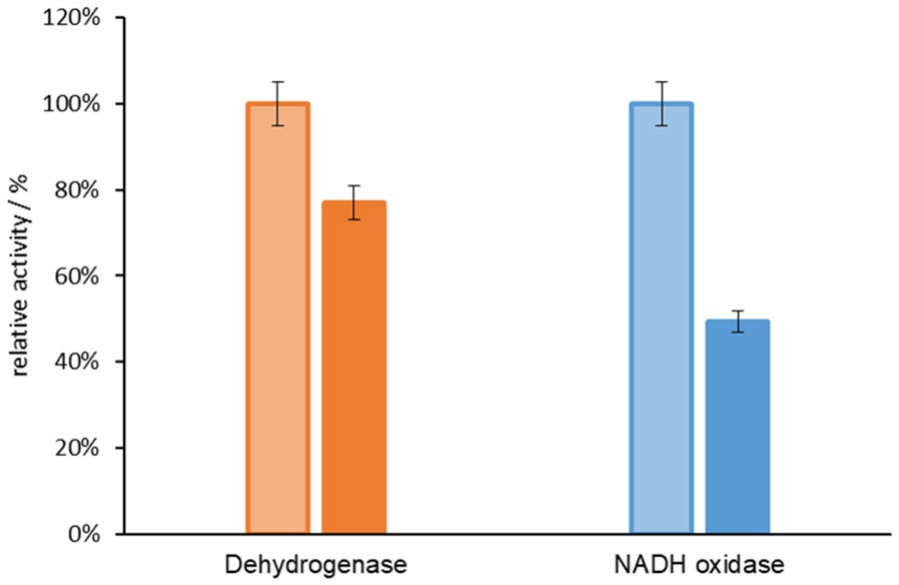
Thermal stability diagram of GldA (orange bar) and SpNox^K184R^ (blue bar)

And then we conducted an experiment on the thermal stability of the enzyme, and the results are shown in Fig. 4. In potassium phosphate buffer (300 mM, pH 7.1), GldA and SpNox^K184R^ still maintained more than 50% activity after 25.5 h at 30 °C.

**Fig. 5:**
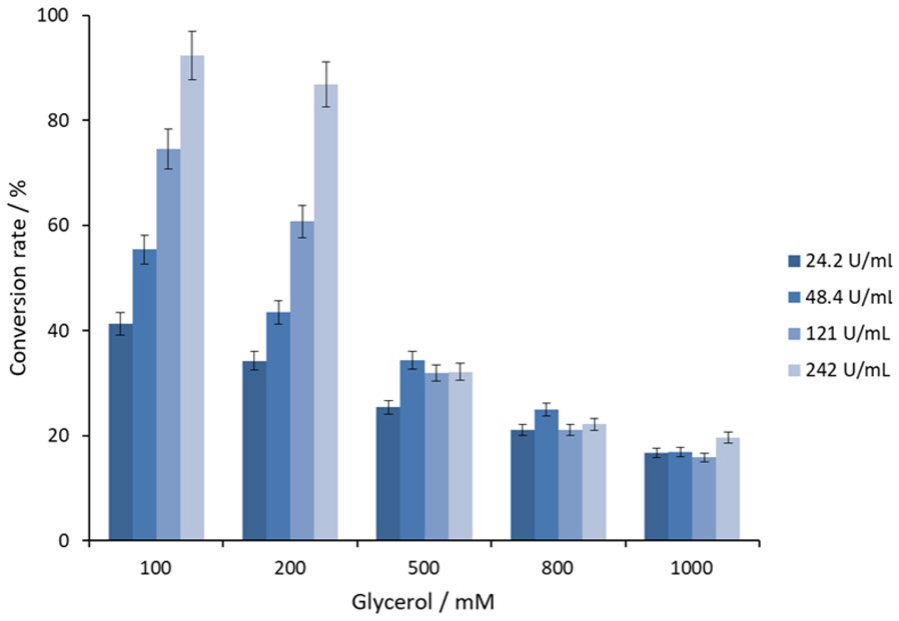
The conversion rate of DHA using GldA-SpNox^K184R^ coupled system. Legend means the amount of GldA added

Finally, the optimal concentration of GldA-SpNox^K184R^ coupled system and substrate glycerol was investigated to achieve higher yield of DHA. In the presence of 2 mM NAD^+^, the yield of DHA at the experimental setup of GldA (24.2–242 U mL^−1^), SpNox^K184R^ (24.2–242 U mL^−1^) and glycerol (100–1000 mM) was quantified. As shown in Fig. 5, at pH 7.1, 300 mM potassium phosphate buffer, 30 °C, the highest yield of DHA, 92.3%, was achieved with 100 mM glycerol and 242 U mL^−1^ GldA and 242 U mL^−1^ SpNox^K184R^. To the best of our knowledge, this is the highest yield for enzymatic conversion of glycerol to DHA. After reaction, the enzyme will appear in a state of aggregation, the aggregated enzyme will lose activity. This may be caused by undesired enzyme interactions (Sanchez et al. 2016).

### Chemoenzymatic conversion of glycerol to lactic acid

The conversion of DHA to lactic acid was performed in NaOH aqueous solution based on our previous study (Li et al. 2020). Firstly, the enzymatic production of DHA with 100 mM glycerol as substrate was carried out and subsequently enzymes was removed from the reaction solution. Then, the reaction solution was added into 5 M NaOH solution with a volume ratio of 1:1. The reaction solution was incubated for 8 h (30 °C, 1000 rpm). Finally, the yield of lactic acid was detected by HPLC. DHA in NaOH solution was completely consumed and the major product was lactic acid. The yield of lactic acid from glycerol in the two-step chemoenzymatic system with GldA, SpNox^K184R^ and NaOH as catalysts was 72.3% (Table 1). Using the coupled GldA-SpNox^K184R^ and sodium hydroxide, the chemoenzymatic synthesis of lactic acid from glycerol was successfully established.

**Table 1:** Conversion rate of DHA to lactic acid and glycolic acid

Co-enzyme circulation reaction system	
Glycerol [mM]	100
Y_DHA from glycerol_ [%]	92.3
NaOH-promoted rearrangement reaction system	
Y _lactic acid from DHA_ [%]	78.3
Y _lactic acid from glycerol_ [%]	72.3
NaClO_2_-catalyzed oxidation reaction system	
Y _glycolic acid from DHA_ [%]	84.7
Y _glycolic acid from glycerol_ [%]	78.2

### Chemoenzymatic conversion of glycerol to glycolic acid

The conversion of DHA to glycolic acid was carried out using NaClO_2_ as oxidant. Firstly, the enzymatic conversion of glycerol to DHA with 100 mM glycerol as substrate was carried out. Subsequently, enzymes were removed from the reaction solution by ultrafiltration. Then, NaClO_2_ solution (four times of the concentration of DHA) was supplemented to the reaction system. The reaction mixture was incubated for 24 h (50 °C, 1000 rpm) and the yield of glycolic acid was detected by HPLC. DHA in NaClO_2_ solution was completely consumed and the major product was glycolic acid. The yield of glycolic acid in the chemoenzymatic system with GldA, SpNox^K184R^ and NaClO_2_ as catalysts was 78.2% (Table 1).

## Conclusions

In summary, two-step chemoenzymatic approaches for the conversion of glycerol into lactic acid and glycolic acid were established and optimized using coupled enzyme system and NaOH/NaClO_2_ with high overall yield. Glycerol-dehydrogenase-GldA from *Escherichia coli* and NADH oxidase-SpNox^K184R^ from *Streptococcus Pyogenes* were used to act together to achieve the dynamic balance of NAD^+^ and NADH transformation, and convert glycerol to DHA. In the enzymatic step, a coenzyme recycling system was developed to convert glycerol into dihydroxyacetone (DHA) with a yield of 92.3% in potassium phosphate buffer (300 mM, pH 7.1) containing 100 mM glycerol, 2 mM NAD^+^, GldA and SpNox^K184R^ concentration of 242 U/mL at 30 °C. The conversion of glycerol to DHA by the coenzyme cycle pathway reduced the generation of by-products and accelerated the catalytic efficiency of the enzymes. After the removal of the enzymes, NaOH or NaClO_2_ catalyzes the formation of lactic acid and glycolic acid from DHA. The high yield of lactic acid (72.3%) and glycolic acid (78.2%) verify the benefit of the chemoenzymatic approaches. The chemoenzymatic production of lactic acid and glycolic acid from low-cost glycerol in this study opens a new route for the utilization of low-cost renewable compound.

## Experimental section

### Materials and methods

Glycerol was purchased from Sinopharm (Beijing, China), NAD^+^ was purchased from MesChemExpress (Shanghai, China), 1,3-dihydroxyacetone (DHA) and O-(2,3,4,5,6-pentafluorobenzyl) hydroxylamine hydrochloride (PFBHA) for HPLC derivatization were purchased from Bidepharm (Shanghai, China), lactic acid was purchased from Aladdin (Shanghai, China), sodium chlorite and glycolic acid were purchased from Macklin (Shanghai, China), Acetonitrile was purchased from Innochen (Beijing, China), isopropyl-beta-D-thiogalactoside (IPTG) (inducer, > 99%) was purchased from Solarbio (Beijing, China), Ampicillin was purchased from Inalco (Beijing, China) and Kanamycin was purchased from Aladdin (Shanghai, China). Oligonucleotide syntheses and DNA sequencing analyses were carried out by Genewiz (Tianjin, China). Seamless Cloning Kit was purchased from Beyotime (Shanghai, China). The amount of DNA in cloning experiments was quantified by TECAN microplate reader (Infinite M200, TECAN, Switzerland).

### Construction of the strains of *E. coli *(pET21b-GldA), *E. coli* (pRSFDuet-SpNox^K184R^)

Plasmids and recombinant strains constructed in this study are listed in Additional File 1: Table S1. The GldA gene (Gene ID: CP034658.1) from *E. coli* strain ATCC 98,082 was cloned into T7 promoter-based plasmid pET21b to construct pET21b-GldA and then the heterologous expression was performed with *E. coli*/BL21-Gold (DE3). The SpNox gene from *Streptococcus pyogenes* MGAS10394 with an amino acid substitution K184R was synthesized by GENEWIZ (Suzhou, China) and cloned into the pRSFDuet-1. pRSFDuet-SpNox^K184R^ was expressed in *E. coli*/BL21-Gold (DE3).

### Expression of recombinant strains in shaking flask and protein purification

BL21-Gold (DE3)*-*pET21b-GldA was inoculated into LB liquid medium (5 mL, 100 μg mL^−1^ ampicillin) and cultivated at 37 °C overnight. BL21-Gold (DE3)-pRSFDuet-SpNox^K184R^ was inoculated into LB liquid medium (5 mL, 50 μg mL^−1^ kanamycin) and cultivated at 37 °C overnight. The precultures were transferred into 700 mL LB liquid medium with the ratio of 1:100, respectively. All the flasks were shaken at 200 rpm at 37 °C until the optical density at 600 nm (OD_600_) reached 0.6–0.8, the cultures were induced by adding 0.5 mM IPTG at 20 °C for 24 h. Subsequently, *E. coli* cells were harvested by centrifugation (4 °C, 6000 rpm, 15 min), resuspended in phosphate buffer (50 mL, 50 mM K_2_HPO_4_ and KH_2_PO_4,_ pH 7.1), and then disrupted with a low temperature ultra-high pressure continuous-flow cell disrupter (LN-3000plus; JNBIO, Guangzhou, China). The disrupted cells were centrifuged (4 °C, 8000 rpm, 20 min), and the supernatants were further filtered through millipore express membrane filters (0.22 μm; Sterile Millex Filter Unit; Merck kGaA, Darmstadt, Germany). The supernatant was loaded onto a nickel affinity column equilibrated with 30 mL nickel sulfate. The GldA was step-wise eluted using elution buffer (50 mM NaH_2_PO_4_, 300 mM NaCl, pH7.4) containing 20, 50, 90, 150, 250 mM imidazole. The SpNox^K184R^ was step-wise eluted using elution buffer (50 mM NaH_2_PO_4_, 300 mM NaCl, pH 7.4) containing 10, 30, 90, 150, 250 mM imidazole. The concentration of protein was determined using a protein assay kit (Micro BCA Protein Assay Kit; Sangon Biotech, Shanghai, China) with BSA as the standard. The purity of the purified protein was assessed by sodium dodecyl sulfate polyacrylamide gel electrophoresis (SDS-PAGE) by using standard molecular biology techniques. SDS-PAGE of GldA and SpNox^K184R^ is shown in Additional File 1: Figure S1.

### Preparation of standard curve of NADH

A standard solution of NADH was prepared, diluted with ddH_2_O, and the absorbance was measured at 340 nm. The linear range was observed to be 0–1 mM. The standard curve of NADH is shown in Additional File 1: Figure S2.

### Enzyme activity detection

The activity of GldA was assayed using purified enzyme (20 μg mL^−1^) at 30 °C in potassium phosphate buffer (300 mM K_2_HPO_4_ and KH_2_PO_4_, pH 7.1) containing 2 mM NAD^+^ and 100 mM glycerol. SpNox^K184R^ was assayed using purified enzyme (25 μg mL^−1^) at 30 °C in potassium phosphate buffer (300 mM K_2_HPO_4_ and KH_2_PO_4_, pH 7.1) containing 2 mM NADH. The increase and decrease of absorbance at 340 nm was detected for 20 min by a microtiter plate reader. One unit of enzyme activity was defined as the amount of enzyme required to produce 1 μmol of NADH per min (GldA). One unit of enzyme activity was defined as the amount of enzyme required to oxidize 1 μmol of NADH per min (SpNox^K184R^). The data are average of two independent experiments (triplicates in each experiment).

Enzyme activity calculation formula:

1 $${\text{U}} = \frac{{{\text{D}} \times 10^{3} \times \left( {\Delta A_{1} - \Delta A_{2} } \right) \times V_{t} }}{{e \times V_{s} \times d \times c}}$$

where D: dilution rate; *∆A*_*1*_: change in NADH absorbance; *∆A*_*2*_: change in absorbance value of blank control; *V*_*t*_: the total reaction system, mL; *e*: molar absorbance, 6.22 × 10^–3^ mol L^−1^ cm^−2^; *V*_*s*_: enzyme product, mL; *d*: optical path distance, cm; and *c*: protein concentration, mg mL^−1^.

Transforming the formula according to the actual requirements:

2 $${\text{U}} = \frac{{\left( {C_{NADH} \times V_{t} } \right)/\Delta T}}{{c \times V_{s} }}$$

where *ΔT* is the reaction time.

According to the standard curve of NADH, the generation of NADH (GldA) or the consumption of NADH (SpNox^K184R^) were calculated and substituted into the formula to finally calculate the enzyme activity.

### Optimization of reaction buffer

Three kinds of buffer solutions (pH 7.1) were investigated as following: potassium phosphate buffer (100 mM K_2_HPO_4_ and KH_2_PO_4_, pH 7.1), sodium phosphoric buffer (100 mM NaH_2_PO_4_ and Na_2_HPO_4_, pH 7.1), Tris–HCl buffer (100 mM Tris–HCl, pH 7.1). GldA activity was assayed using purified enzyme (20 μg mL^−1^) at 30 °C in different buffer containing 2 mM NAD^+^ and 100 mM glycerol. SpNox^K184R^ was assayed using purified enzyme (25 μg mL^−1^) at 30 °C in different buffer containing 2 mM NADH. The increase and decrease of absorbance at 340 nm was detected for 20 min by a microtiter plate reader. One unit of enzyme activity was defined as the amount of enzyme required to produce 1 μmol of NADH per min (GldA). One unit of enzyme activity was defined as the amount of enzyme required to oxidize 1 μmol of NADH per min (SpNox^K184R^). The data are average of three independent experiments (triplicates in each experiment). The optimum buffer was selected according to the activity of the two enzymes.

### Optimization of the pH value and ionic strength of the buffer

According to the characteristics of the enzyme, pH 6.1–7.1 was selected as the enzyme activities were detected in different pH buffers. GldA activity was assayed using purified enzyme (20 μg mL^−1^) at 30 °C in different buffer containing 2 mM NAD^+^ and 100 mM glycerol. SpNox^K184R^ was assayed using purified enzyme (25 μg mL^−1^) at 30 °C in different buffer containing 2 mM NADH. The increase and decrease of absorbance at 340 nm was detected for 20 min by a microtiter plate reader. One unit of enzyme activity was defined as the amount of enzyme required to produce 1 μmol of NADH per min (GldA). One unit of enzyme activity was defined as the amount of enzyme required to oxidize 1 μmol of NADH per min (SpNox^K184R^). The data are average of three independent experiments (triplicates in each experiment). The optimum buffer was selected according to the activity of the two enzymes. After selecting pH and buffer type, the buffer concentration was optimized. 20–500 mM potassium phosphate buffers (K_2_HPO_4_ and KH_2_PO_4_) were prepared at pH 7.1. GldA activity was assayed using purified enzyme (20 μg mL^−1^) at 30 °C in different buffer containing 2 mM NAD^+^ and 100 mM glycerol. SpNox^K184R^ was assayed using purified enzyme (25 μg mL^−1^) at 30 °C in different buffer containing 2 mM NADH. The increase and decrease of absorbance at 340 nm was detected for 20 min by a microtiter plate reader. One unit of enzyme activity was defined as the amount of enzyme required to produce 1 μmol of NADH per min (GldA). One unit of enzyme activity was defined as the amount of enzyme required to oxidize 1 μmol of NADH per min (SpNox^K184R^). The data are average of three independent experiments (triplicates in each experiment). The optimum buffer was selected according to the activity of the two enzymes.

### Enzymatic stability of GldA and SpNox^K184R^

In potassium phosphate buffer (300 mM, pH 7.1), GldA and SpNox^K184R^ were stored at 30 °C for 25.5 h. Then the enzyme activity was detected and compared with the original enzyme activity to determine the stability of the enzyme.

### Biooxidation of glycerol to DHA by GldA coupled with SpNox^K184R^

The ratio of GldA and SpNox^K184R^ was adjusted according to the enzyme specific activity, with activity ratio of 1:1. The 500 μL reaction mixture in 30 °C, potassium phosphate buffer (300 mM K_2_HPO_4_ and KH_2_PO_4_, pH7.1) containing 100 mM–1 M glycerol, 2 mM NAD^+^, 24.2–242 U mL^−1^ GldA, SpNox^K184R^ was the same enzyme activity as GldA (the enzyme activity ratio of GldA and SpNox^K184R^ is 1:1). The reactions were carried out in 4.5 mL tubes at 30 °C with shaking (1000 rpm) for 14 h.

### The high-performance liquid chromatography (HPLC) analysis for DHA

The HPLC method was used for the detection of DHA. After removing the enzymes using the concurrent filter unit (Millipore UFC500 3 kDa Filter Devices, The United States) through ultrafiltration at 8000 rpm for 20 min, 30 μL sample solution was added in 1.5 mL microcentrifuge tube containing 140 μL 20 mg mL^−1^ PFBHA solution (PFBHA was dissolved into citric acid buffer (0.1 M, citric acid and NaOH, pH 4) and adjusted to pH 4.0 with 1 M NaOH), 30 μL 50 mM veratryl alcohol (internal standard). After reaction at 1000 rpm, 30 °C for 30 min, 200 μL acetonitrile and 100 μL water were added in the tube. The samples were further filtered through 0.22 μm organic filtration membrane and was used for HPLC detection. Detection conditions after derivatization by HPLC (Thermo, UltiMate 3000): Ultimate XB-C18 column, 4.6 × 250 mm, 5 μm; mobile phase: water, acetonitrile; UV absorption wavelength: 263 nm; the flow rate: 1.2 mL min^−1^; column temperature: 30 °C; sample injection volume: 20 μL. The standard curve of DHA is shown in Additional File 1: Figure S3.

### The synthesis of lactic acid from glycerol

Firstly, glycerol was converted to DHA using the coenzyme circulation system (500 μL) including 100 mM glycerol, 2 mM NAD^+^, 242 U mL^−1^ GldA and 242 U mL^−1^ SpNoX^K184R^ and 300 mM potassium phosphate buffer (pH7.1). At the end of the reaction, the enzymes were removed and injected slowly into 500 μL NaOH (5 M) through the channel injection pump with a syringe at a rate of 100 μL h^−1^. The reaction was conducted for 8 h (1000 rpm, 30 °C). After 8 h, reaction mixture was acidified with dilute sulfuric acid to pH 1–2 for HPLC analysis. The HPLC system was carried out on an Agilent 1260 system equipped with a UV detector (210 nm), and fitted with Sugar 10H column (DEVOTE): mobile phase: 5 mM H_2_SO_4_, flow rate: 0.5 mL min^−1^, sample volume: 20 μL, column temperature: 35 °C. Triplicates in each experiment. The standard curve of lactic acid is shown in Additional File 1: Figure S4.

### The synthesis of glycolic acid from glycerol

Firstly, glycerol was converted to DHA using the coenzyme circulation system (500 μL) including 100 mM glycerol, 2 mM NAD^+^, 242 U mL^−1^ GldA and 242 U mL^−1^ SpNoX^K184R^ and 300 mM potassium phosphate buffer (pH7.1). The 500 μL reaction solution was added into 1.5 mL microcentrifuge tube containing 500 μL NaClO_2_ solution (the concentration of NaClO_2_ solution is about 4 times concentration of DHA). The reaction was conducted for 24 h (1000 rpm, 50 °C). After, the glycolic acid production was detected by HPLC directly. The HPLC system was carried out on an Agilent 1260 system equipped with a UV detector (210 nm), and fitted with Sugar 10H column (DEVOTE): mobile phase: 5 mM H_2_SO_4_, flow rate: 0.5 mL min^−1^, sample volume: 20 μL, column temperature: 35 °C. Triplicates in each experiment. The standard curve of glycolic acid is shown in Additional File 1: Figure S5.

### Yield calculation

The yields of all products were calculated with the following equations:

3 $${\text{Yield}}_{{\left( {{\text{DHA}}\,{\text{from}}\,{\text{glycerol}}} \right)}} = \frac{{{\text{produced}}\,{\text{DHA}}}}{{{\text{glycerol}}}} \times 100\% ,$$

4 $${\text{Yield}}_{{\left( {{\text{lactic}}\,{\text{acid}}\,{\text{from}}\,{\text{DHA}}} \right)}} = \frac{{{\text{produced}}\,{\text{lactic}}\,{\text{acid}}}}{{{\text{generated}}\,{\text{DHA}}}} \times 100\% ,$$

5 $${\text{Yield}}_{{\left( {{\text{lactic}}\,{\text{acid}}\,{\text{from}}\,{\text{glycerol}}} \right)}} = \frac{{{\text{produced}}\,{\text{lactic}}\,{\text{acid}}}}{{{\text{glycerol}}}} \times 100\% ,$$

6 $${\text{Yield}}_{{\left( {{\text{glycolic}}\,{\text{acid}}\,{\text{from}}\,{\text{DHA}}} \right)}} = \frac{{{\text{produced}}\,{\text{glycolic}}\,{\text{acid}}}}{{{\text{generated}}\,{\text{DHA}}}} \times 100\% ,$$

$${\text{Yield}}_{{\left( {{\text{glycolic}}\,{\text{acid}}\,{\text{from}}\,{\text{glycerol}}} \right)}} = \frac{{{\text{produced}}\,{\text{glycolic}}\,{\text{acid}}}}{{{\text{glycerol}}}} \times 100\%$$

HPLC analysis of chemoenzymatic conversion of glycerol to lactic acid and glycolic acid is shown in Additional File 1: Figure S6.

### Supplementary Information

**Additional file 1:** **Table S1.** The Plasmids and recombinant strains. **Figure S1.** SDS-PAGE of glycerol dehydrogenase (GldA) and NADH oxidase (SpNox^K184R^). (M: Prestained Protein Ladder, 1: Crude GldA, 2: Purified GldA, 3: Crude SpNox^K184R^, 4: Purified SpNoxK^184R^). **Figure S2.** Calibration curve of 96-well microtiter-plate-format NADH colorimetric screening assay. Aqueous solutions of 0–1 mM NADH were successively added to a 96-well microtitration plate. The absorbance value of NADH was detected by a microplate reader at 340 nm. The standard curve of NADH concentration was calculated under the condition of subtracting background. **Figure S3.** HPLC chromatogram and standard curve of 1,3-Dihydroxyacetone (DHA). (a) HPLC chromatogram of derived DHA. The number 1 is internal standard veratryl alcohol, the number 2 is the standard 1,3-Dihydroxyacetone (DHA) and the number 3 is the derivative O-(2,3,4,5,6-Pentafluorobenzyl) (PFBHA). (b) The standard curve of DHA. Notes: Detection conditions after derivatization by using HPLC (Thermo, UltiMate 3000): Ultimate XB-C18 column, 4.6×250 mm, 5 μm; mobile phase: water, acetonitrile; UV absorption wavelength: 263 nm; flow rate: 1.2 mL min-1; column temperature: 30 °C; sample injection volume: 20 μL. The standard curve was calculated according to the peak area of DHA detected by HPLC. **Figure S4.** HPLC chromatogram and standard curve of lactic acid. (a) HPLC chromatogram of lactic acid. The peak of lactic acid is labelled. (b) The standard curve of lactic acid. Notes: The HPLC analysis was carried out on an Agilent 1260 system equipped with a UV detector (210 nm), and fitted with Sugar 10H column (DEVOTE) : mobile phase: 5 mM H_2_SO_4_, flow rate: 0.5 mL min-1, column temperature: 35 °C, sample volume: 20 μL. The standard curve was calculated according to the peak area of lactic acid detected by HPLC. **Figure S5.** HPLC chromatogram and standard curve of glycolic acid. (a) HPLC chromatogram of glycolic acid. The peak of glycolic acid is labelled. (b) The standard curve of glycolic acid. Notes: The HPLC system was carried out on an Agilent 1260 system equipped with a UV detector (210 nm), and fitted with Aminex HPX- 87H column (DEVOTE): mobile phase: 5 mM H_2_SO_4_, flow rate: 0.5 mL min-1, column temperature: 35 °C, sample volume: 20 μL. The standard curve was calculated according to the peak area of glycolic acid detected by HPLC. **Figure S6.** HPLC chromatogram of the conversion of glycerol to lactic acid and glycolic acid, respectively. (a) The synthesis of DHA from glycerol catalyzed by coenzyme system was determined by derivation method. The number 1 is the internal standard veratryl alcohol, the number 3 is DHA standard and the number 4 is the derivative O-(2,3,4,5,6-Pentafluorobenzyl) (PFBHA). DHA was measured by a derivative method. 100 mM glycerol was catalyzed into DHA by a coenzyme cycle at 30 °C for 14 h. (b) HPLC chromatogram of lactic acid generated from the conversion of DHA (glycerol catalyzed by coenzyme cycle system) in the solution of NaOH. The number 1 is lactic acid, the number 2 is DHA. The black line represents reaction in NaOH solution, the red line represents the control without DHA. (c) HPLC chromatogram of glycolic acid produced from DHA in NaClO_2_ solution. The number 1 is glycolic acid, the number 2 is DHA. The black line represents the reaction catalyzed in NaClO_2_ solution, the red line represents the control without DHA.

## Data Availability

All data supporting this article’s conclusion are available.

## References

[CR1] Evans WL, Cornthwaite WR (2002). The mechanism of carbohydrate oxidation. VII. the action of potassium hydroxide on dihydroxy acetone. J Am Chem Soc.

[CR2] Hasegawa S, Azuma M, Takahashi K (2008). Stabilization of enzyme activity during the esterification of lactic acid in hydrophobic ethers and ketones as reaction media that are miscible with lactic acid despite their high hydrophobicity. Enzyme Microb Technol.

[CR3] Huang J, Wei J, Jin S, Zou Q, Li J, Zuo Y, Li Y (2020). The ultralong-term comparison of osteogenic behavior of three scaffolds with different matrices and degradability between one and two years. J Mater Chem B.

[CR4] Huang S, Xue Y, Yu B, Wang L, Zhou C, Ma Y (2021). A review of the recent developments in the bioproduction of polylactic acid and its precursors optically pure lactic acids. Molecules.

[CR5] Kosamia NM, Samavi M, Uprety BK, Rakshit SK (2020). Valorization of biodiesel byproduct crude glycerol for the production of bioenergy and biochemicals. Catalysts.

[CR6] Lari G, Mondelli C, Perez Ramirez J (2015). Gas-phase oxidation of glycerol to dihydroxyacetone over tailored iron zeolites. ACS Catal.

[CR7] Lasprilla AJR, Martinez GAR, Lunelli BH, Jardini AL, Filho RM (2012). Poly-lactic acid synthesis for application in biomedical devices—a review. Biotechnol Adv.

[CR8] Li T, Tang Z, Wei H, Tan Z, Liu P, Li J, Zheng Y, Lin J, Liu W, Jiang H, Liu H, Zhu L, Ma Y (2020). Totally atom-economical synthesis of lactic acid from formaldehyde: combined bio-carboligation and chemo-rearrangement without the isolation of intermediates. Green Chem.

[CR9] Liu Z, Jia L, Zheng Y (2010). Biotransformation of DL-lactate to pyruvate by a newly isolated Serratia marcescens ZJB-07166. Process Biochem.

[CR10] Liu D, Liu J, Cai W, Ma J, Yang H, Xiao H, Li J, Xiong Y, Huang Y, Liu B (2019). Selective photoelectrochemical oxidation of glycerol to high value-added dihydroxyacetone. Nat Commun.

[CR11] López-Gómez JP, Alexandri M, Schneider R, Venus J (2019). A review on the current developments in continuous lactic acid fermentations and case studies utilising inexpensive raw materials. Process Biochem.

[CR12] Luo D, Yin W, Han D, He H, Xia S (2021). Glycolic acid and formic acid production from pyrolysis oil water-soluble fraction by catalytic oxidation. Chem Eng Sci.

[CR13] Lux S, Siebenhofer M (2013). Synthesis of lactic acid from dihydroxyacetone: use of alkaline-earth metal hydroxides. Catal Sci Technol.

[CR14] Marianou AA, Michailof CC, Ipsakis D, Triantafyllidis K, Lappas AA (2019). Cellulose conversion into lactic acid over supported HPA catalysts. Green Chem.

[CR15] Niu W, Kramer L, Mueller J, Liu K, Guo J (2019). Metabolic engineering of Escherichia coli for the de novo stereospecific biosynthesis of 1,2-propanediol through lactic acid. Metab Eng Commun.

[CR16] Oscar V, Jan EB (2015). Chemoenzymatic dynamic kinetic resolution: a powerful tool for the preparation of enantiomerically pure alcohols and amines. J Am Chem Soc.

[CR17] Oude Elferink SJWH, Krooneman J, Gottschal JC, Spoelstra SF, Faber F, Driehuis F (2001). Anaerobic conversion of lactic acid to acetic acid and 1,2-propanediol by *Lactobacillus buchneri*. Appl Environ Microbiol.

[CR18] Piattoni CV, Figueroa CM, Diez MDA, Parcerisa IL, Antuña S, Comelli RA, Guerrero SA, Beccaria AJ, Iglesias AÁ (2013). Production and characterization of Escherichia coli glycerol dehydrogenase as a tool for glycerol recycling. Process Biochem.

[CR19] Pullanikat P, Jung SJ, Yoo KS, Jung KW (2010). Oxidative degradation of reducing carbohydrates to ammonium formate with H2O2 and NH4OH. Tetrahedron Lett.

[CR20] Sanchez A, Cruz J, Rueda N, dos Santos JCS, Torres R, Ortiz C, Villalonga R, Fernandez-Lafuente R (2016). Inactivation of immobilized trypsin under dissimilar conditions produces trypsin molecules with different structures. RSC Adv.

[CR21] Shi YH, Sun HY, Lu DM, Le QH, Chen DX, Zhou YC (2006). Separation of glycolic acid from glycolonitrile hydrolysate by reactive extraction with tri-n-octylamine. Sep Purif Technol.

[CR22] Shi H, Yin X, Bala S, Raghunath VC (2019). Liquid-phase oxidation of ethylene glycol on Pt and Pt–Fe catalysts for the production of glycolic acid: remarkable bimetallic effect and reaction mechanism. Ind Eng Chem Res.

[CR23] Shi Q, Guo H, Chen C, Hou B, Jia L, Li D (2020). An efficient Bronsted acidic polymer resin for the carbonylation of formaldehyde to glycolic acid. React Kinet Mech Catal.

[CR24] Shi Q, Guo H, Chen C, Hou B, Jia L, Li D (2020). An efficient Brønsted acidic polymer resin for the carbonylation of formaldehyde to glycolic acid. React Kinet Mech Catal.

[CR25] Singh S, Ahuja K (2018) Glycerol market size. https://www.gminsights.com/industryanalysis/glycerol-market-size

[CR26] Sotto N, Cazorla C, Villette C (2016). Toward the sustainable synthesis of biosourced divinylglycol from glycerol. Acs Sustain Chem Eng.

[CR27] Sun D, Yamada Y, Sato S, Ueda W (2017). Glycerol as a potential renewable raw material for acrylic acid production. Green Chem.

[CR28] Tshibalonza NN, Monbaliu JCM (2017). Revisiting the deoxydehydration of glycerol towards allyl alcohol under continuous-flow conditions. Green Chem.

[CR29] Veluturla S, Archna N, Subba Rao D, Hezil N, Indraja IS, Spoorthi S (2018). Catalytic valorization of raw glycerol derived from biodiesel: a review. Biofuels UK.

[CR30] Walgode PM, Faria RPV, Rodrigues AE (2020). A review of aerobic glycerol oxidation processes using heterogeneous catalysts: a sustainable pathway for the production of dihydroxyacetone. Catal Rev Sci Eng.

[CR31] Wang J, HannahJiangNingFangLiu LSYMS (2020). Optimization of immobilized *Lactobacillus pentosus* cell fermentation for lactic acid production. Biores Bioprocess.

[CR32] Wang H, Yu D, Sun P, Yan J, Wang Y, Huang H (2008). Rare earth metal modified NaY: structure and catalytic performance for lactic acid dehydration to acrylic acid. Catal Commun.

[CR33] Xu G, Ni Y (2015). Bioreductive preparation of ACE inhibitors precursor (R)-2-hydroxy-4-phenylbutanoate esters: recent advances and future perspectives. Biores Bioprocess.

[CR34] Xu P, Zheng G-W, Zong M-H, Li N, Wen-Yong L (2017). Recent progress on deep eutectic solvents in biocatalysis. Biores Bioprocess.

[CR35] Xu S, Zhang L, Zhou S, Deng Y (2021). Biosensor-based multi-gene pathway optimization for enhancing the production of glycolate. Appl Environ Microbiol.

[CR36] Yun Eun J, Zhang GC, Atkinson C, Lane S, Liu JJ, Ort Donald R, Jy Su (2021). Glycolate production by a *Chlamydomonas reinhardtii* mutant lacking carbon-concentrating mechanism. J Biotechnol.

[CR37] Zhang J, Li X, Pang J, Zou W, Tang C, Dong L (2019). An efficient and durable hierarchically porous KLA/TiPO catalyst for vapor phase condensation of lactic acid to 2,3-pentanedione. New J Chem.

[CR38] Zhu Z, Kang G, Yu S, Qin Y, Sun Y, Cao Y (2020). Process intensification in carbonylation of formaldehyde with active and passive enhancement methods. J Flow Chem.

[CR39] Zhu T, Yao D, Li D, Xu H, Jia S, Bi C, Cai J, Zhu X, Zhang X (2021). Multiple strategies for metabolic engineering of Escherichia coli for efficient production of glycolate. Biotechnol Bioeng.

