# Supplementary material for: Chemoenzymatic conversion of glycerol to lactic acid and glycolic acid
Source: Bioresour Bioprocess. 2022 Jul 18;9(1):75. doi: 10.1186/s40643-022-00561-z (PMC10992446; doi:10.1186/s40643-022-00561-z)
Supplement: Supplementary file 1 — Additional file 1: Table S1. The Plasmids and recombinant strains. Figure S1. SDS-PAGE of glycerol dehydrogenase (GldA) and NADH oxidase (SpNoxK184R). (M: Prestained Protein Ladder, 1: Crude GldA, 2: Purified GldA, 3: Crude SpNoxK184R, 4: Purified SpNoxK184R). Figure S2. Calibration curve of 96-well microtiter-plate-format NADH colorimetric screening assay. Aqueous solutions of 0–1 mM NADH were successively added to a 96-well microtitration plate. The absorbance value of NADH was detected by a microplate reader at 340 nm. The standard curve of NADH concentration was calculated under the condition of subtracting background. Figure S3. HPLC chromatogram and standard curve of 1,3-Dihydroxyacetone (DHA). (a) HPLC chromatogram of derived DHA. The number 1 is internal standard veratryl alcohol, the number 2 is the standard 1,3-Dihydroxyacetone (DHA) and the number 3 is the derivative O-(2,3,4,5,6-Pentafluorobenzyl) (PFBHA). (b) The standard curve of DHA. Notes: Detection conditions after derivatization by using HPLC (Thermo, UltiMate 3000): Ultimate XB-C18 column, 4.6×250 mm, 5 μm; mobile phase: water, acetonitrile; UV absorption wavelength: 263 nm; flow rate: 1.2 mL min-1; column temperature: 30 °C; sample injection volume: 20 μL. The standard curve was calculated according to the peak area of DHA detected by HPLC. Figure S4. HPLC chromatogram and standard curve of lactic acid. (a) HPLC chromatogram of lactic acid. The peak of lactic acid is labelled. (b) The standard curve of lactic acid. Notes: The HPLC analysis was carried out on an Agilent 1260 system equipped with a UV detector (210 nm), and fitted with Sugar 10H column (DEVOTE) : mobile phase: 5 mM H2SO4, flow rate: 0.5 mL min-1, column temperature: 35 °C, sample volume: 20 μL. The standard curve was calculated according to the peak area of lactic acid detected by HPLC. Figure S5. HPLC chromatogram and standard curve of glycolic acid. (a) HPLC chromatogram of glycolic acid. The peak of glycolic acid is labelled. (b [file 40643_2022_561_MOESM1_ESM.pdf]

Supporting Information

Chemoenzymatic conversion of glycerol to lactic acid and glycolic acid

Yue Ma[1,2], Tianzhen Li[2], Long Ma[1], Haifeng Liu[3]* and Leilei Zhu[2]*

[1]State Key Laboratory of Food Nutrition and Safety, College of Biotechnology, Tianjin University of Science and Technology, Tianjin 300457, China

[2]Tianjin Institute of Industrial Biotechnology, Chinese Academy of Sciences; National Technology Innovation Center of Synthetic Biology, Tianjin, 300308, China

[3]Jiangsu Collaborative Innovation Centre of Chinese Medicinal Resources Industrialization, School of Pharmacy Nanjing University of Chinese Medicine, Nanjing, Jiangsu, 210023, China

Corresponding Author: haifeng.liu@njucm.edu.cn; zhu_ll@tib.cas.cn.

Table of Contents

Results ········· 3

Table S1 ········· 3

Figure S1 ········· 3

Figure S2 ········· 3

Figure S3 ········· 4

Figure S4 ········· 5

Figure S5 ········· 6

Figure S6 ········· 7

Results

Table S1. The Plasmids and recombinant strains.

Plasmid	Oligonucleotide primers
pET21b-GldA	F: 5'-GGAGATATACATATGGACCGCATTATTCAATCAC-3'
	R: 5'-CTGCGCTAGTAGACCTTTCGGGCTTTGTTAGC-3'
pRSFDuet-SpNox^{K184R}	F: 5'-GCCCGAAAGGTCTACTAGCGCAGCTTAATTAA-3'
	R: 5'-GCGGTCCATATGTATATCTCCTTCTTATACTTAAC-3'

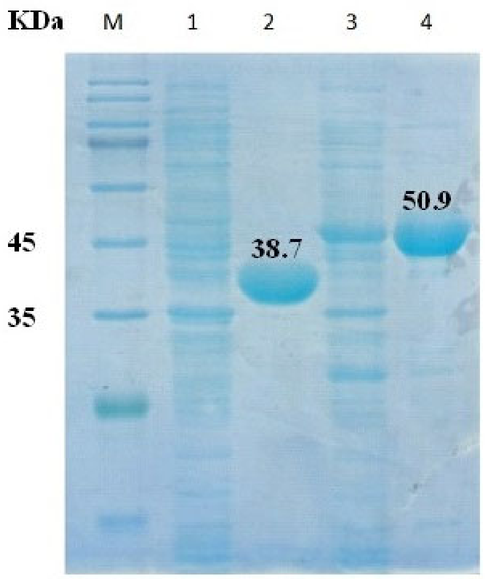

Figure S1. SDS-PAGE of glycerol dehydrogenase (GldA) and NADH oxidase (SpNox^{K184R}). (M: Prestained Protein Ladder, 1: Crude GldA, 2: Purified GldA, 3: Crude SpNox^{K184R}, 4: Purified SpNox^{K184R})

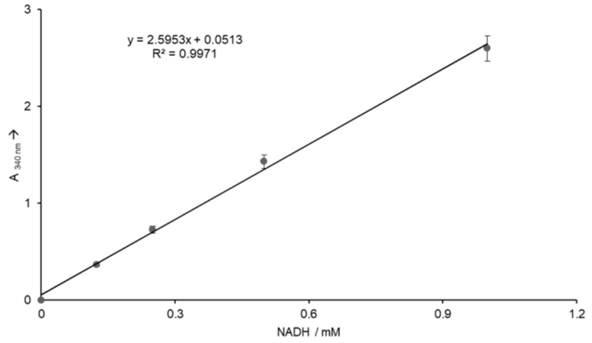

Figure S2. Calibration curve of 96-well microtiter-plate-format NADH colorimetric screening assay. Aqueous solutions of 0-1mM NADH were successively added to a 96-well microtitration plate. The absorbance value of NADH was detected by a microplate reader at 340 nm. The standard curve of NADH concentration was calculated under the condition of subtracting background.

SUPPLEMENTARY FIGURES AND TABLES

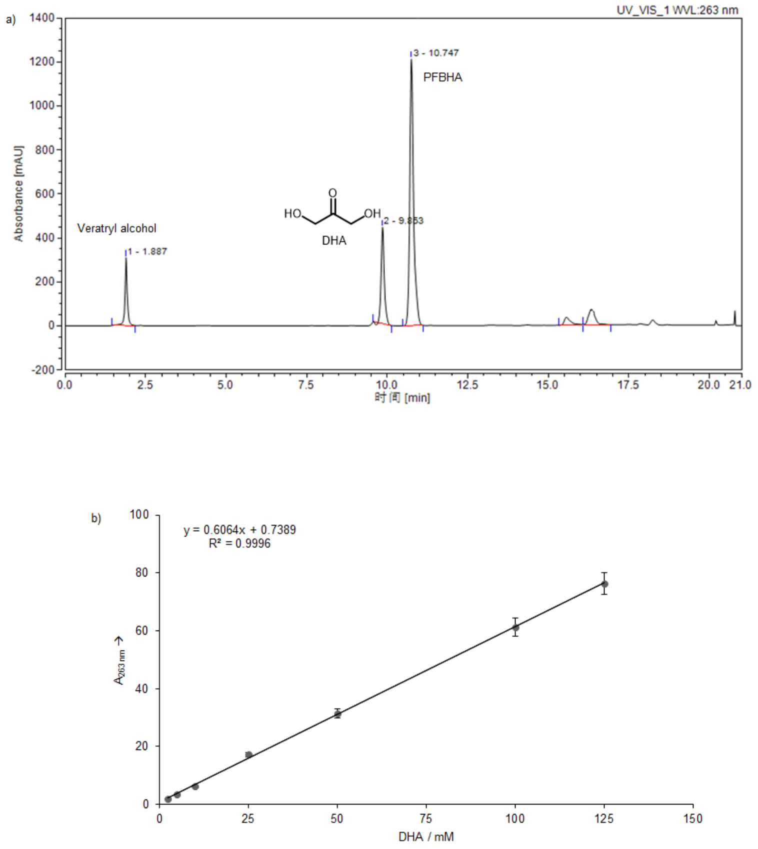

Figure S3. HPLC chromatogram and standard curve of 1,3-Dihydroxyacetone (DHA). a) HPLC chromatogram of derived DHA. The number 1 is internal standard veratryl alcohol, the number 2 is the standard 1,3-Dihydroxyacetone (DHA) and the number 3 is the derivative O-(2,3,4,5,6-Pentafluorobenzyl) (PFBHA). b) The standard curve of DHA. Notes: Detection conditions after derivatization by using HPLC (Thermo, UltiMate 3000): Ultimate XB-C18 column, 4.6×250 mm, 5 μm; mobile phase: water, acetonitrile; UV absorption wavelength: 263 nm; flow rate: 1.2 mL min^{-1}; column temperature: 30°C; sample injection volume: 20 μL. The standard curve was calculated according to the peak area of DHA detected by HPLC.

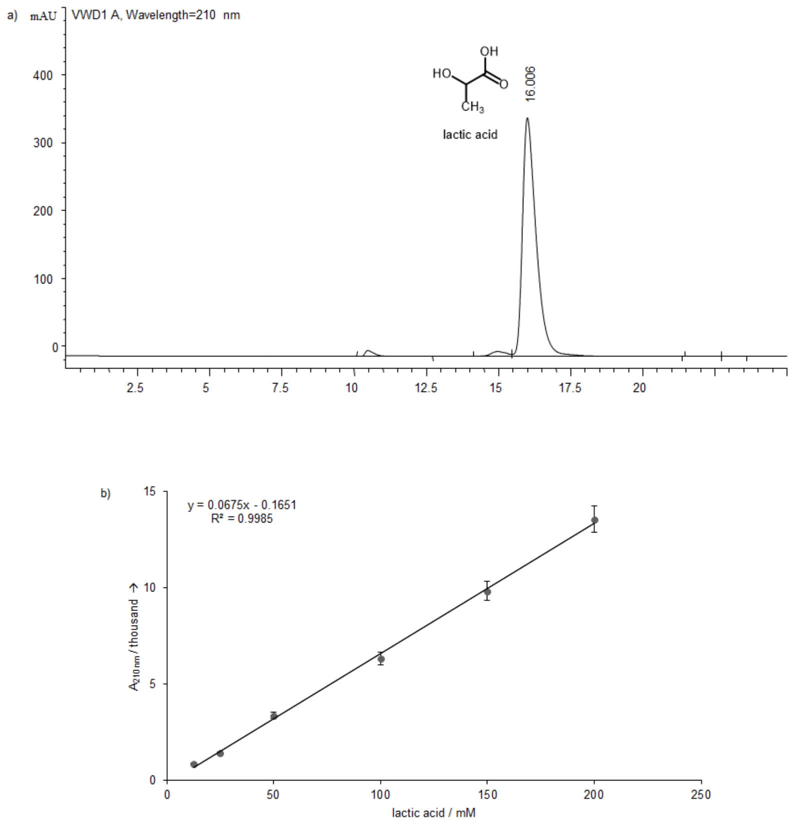

Figure S4. HPLC chromatogram and standard curve of lactic acid**.** a) HPLC chromatogram of lactic acid. The peak of lactic acid is labelled. b) The standard curve of lactic acid. Notes: The HPLC analysis was carried out on an Agilent 1260 system equipped with a UV detector (210 nm), and fitted with Aminex HPX-87H column (DEVOTE) : mobile phase: 5 mM H_2SO_4, flow rate: 0.5 mL min^{-1}, column temperature: 35°C, sample volume: 20 μL. The standard curve was calculated according to the peak area of lactic acid detected by HPLC.

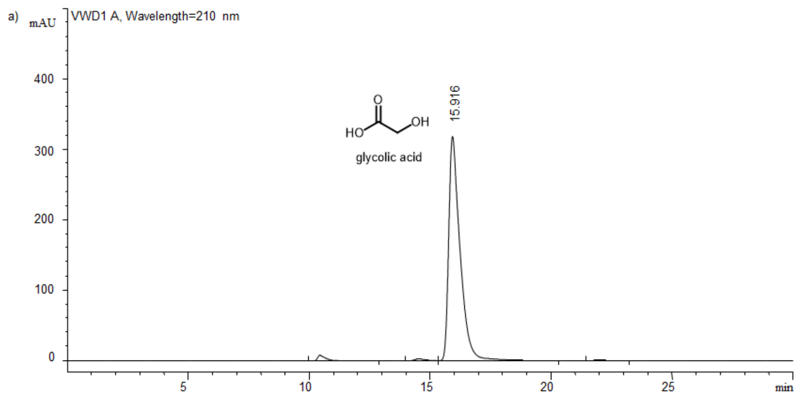

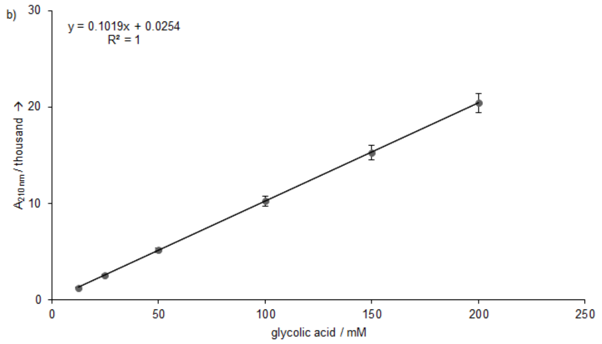

Figure S5. HPLC chromatogram and standard curve of glycolic acid. a) HPLC chromatogram of glycolic acid. The peak of glycolic acid is labelled. b) The standard curve of glycolic acid. **Notes**: The HPLC system was carried out on an Agilent 1260 system equipped with a UV detector (210 nm), and fitted with Aminex HPX-87H column (DEVOTE): mobile phase: 5 mM H_2SO_4, flow rate: 0.5 mL min^{-1}, column temperature: 35°C, sample volume: 20 µL. The standard curve was calculated according to the peak area of glycolic acid detected by HPLC.

SUPPLEMENTARY FIGURES AND TABLES

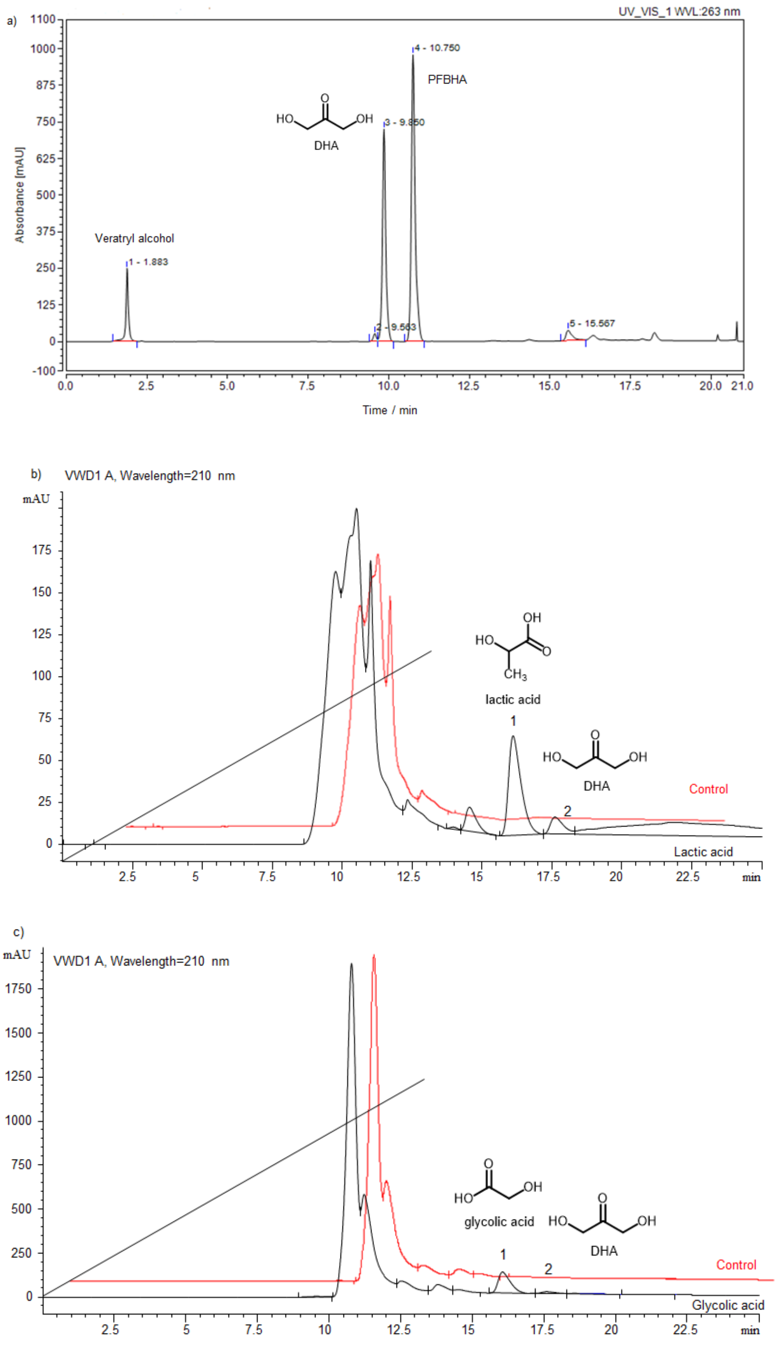

Figure S6. HPLC chromatogram of the conversion of glycerol to lactic acid and glycolic acid, respectively. a) The synthesis of DHA from glycerol catalyzed by coenzyme system was determined by derivation method. The number 1 is the internal standard veratryl alcohol, the number 3 is DHA standard and the number 4 is the derivative O-(2,3,4,5,6-Pentafluorobenzyl) (PFBHA). DHA was measured by a derivative method. 100 mM glycerol was catalyzed into DHA by a coenzyme cycle at 30°C for 14 h. b) HPLC chromatogram of lactic acid generated from the conversion of DHA (glycerol catalyzed by coenzyme cycle system) in the solution of NaOH. The number 1 is lactic acid, the number 2 is DHA. The black line represents reaction in NaOH solution, the red line represents the control without DHA. c) HPLC chromatogram of glycolic acid produced from DHA in $NaClO_2$ solution. The number 1 is glycolic acid, the number 2 is DHA. The black line represents the reaction catalyzed in $NaClO_2$ solution, the red line represents the control without DHA.
